# Ultrafast degradation of azo dyes catalyzed by cobalt-based metallic glass

**DOI:** 10.1038/srep18226

**Published:** 2015-12-14

**Authors:** X. D. Qin, Z. W. Zhu, G. Liu, H. M. Fu, H. W. Zhang, A. M. Wang, H. Li, H. F. Zhang

**Affiliations:** 1 Shenyang National Laboratory for Materials Science, Institute of Metal Research, Chinese Academy of Sciences, Shenyang. 110016, China; 2 University of Chinese Academy of Sciences, Beijing. 100049, China

## Abstract

Reactivity and mass loss are considered mutually exclusive in conventional zero-valent metal (ZVM) technology to treat environmental contaminants. Here, we report the outstanding performance of Co-based metallic glass (MG) in degrading an aqueous solution of azo dye, thus eliminating this trade-off. Ball-milled Co-based MG powders completely degrade Acid Orange II at an ultrafast rate. The surface-area-normalized rate constant of Co-based MG powders was one order of magnitude higher than that of Co-based crystalline counterparts and three orders of magnitude higher than that of the widely studied Fe^0^ powders. The coordinatively unsaturated local structure in Co-based MG responds to the catalysis for degradation, resulting in very low mass loss. Wide applicability and good reusability were also present. Co-based MG is the most efficient material for azo dye degradation reported thus far, and will promote the practical application of MGs as functional materials.

Dyes make the world more colorful but contribute significantly to the water pollution. Synthetic dyes, particularly azo dyes with aromatic structures, are refractory to degradation^1^,^2^, and the threat of dyes to the environment and ecosystem has attracted substantial public attention. Among the various physical and/or chemical processes to treat the wastewater with azo dyes, zero-valent metals (ZVMs)^3^,^4^,^5^,^6^,^7^, including Fe^0^, Mg^0^ and Zn^0^, are of high interest because they are inexpensive, easy to use and non-toxic, and do not require further treatment of effluents. However, two key factors limit their wide practical application^8^,^9^,^10^,^11^. The first is the low reactivity of ZVMs, which results in a limited loading rate unsuitable for the industrial wastewater treatment. The second is the high mass loss of ZVMs due to corrosion in aqueous solution, which contributes to their low durability and induces secondary pollution, obviously increasing the processing cost. Several strategies have been proposed to enhance the reactivity of ZVMs, including making nanostructures by increasing the reactive surface area^11^,^12^,^13^,^14^,^15^, preparing bi-metals^10^,^16^,^17^,^18^,^19^ (Pd-Fe^0^, Ni-Fe^0^, and Pd-Mg^0^, *etc.*) by increasing the oxidation of ZVMs through the formation of infinite galvanic cells simultaneous with facilitating atomic hydrogen reduction by noble metal additives, and coupling using other physical techniques^20^,^21^,^22^,^23^,^24^,^25^, *e.g.*, ultrasound, UV-visible light and microwave. However, these methods enhance reactivity at the cost of intrinsic acceleration of the mass loss of ZVMs^8^,^9^,^11^ and may be more effective in groundwater remediation than industrial wastewater treatment because of the increase in cost and the secondary pollution. Thus, high-performance materials with high reactivity and without the expense of mass loss are urgently needed for industrial wastewater treatment.

Metallic glasses (MGs) show the good combination of some desirable chemical and physical properties such as corrosion resistance, soft magnetic property and high strength^26^,^27^. In addition to structural applications, various functional applications of MGs have also been investigated and exploited^28^,^29^,^30^, including as catalysts, biomaterials, and nanotechnology. Fe-Si-B and Fe-Mo-Si-B MGs were recently reported to decolorize azo dyes at an enhanced surface area normalized degradation rate with compared to crystalline Fe^0^ and nano-Fe^0^ 31,32. These findings develop a tremendous application potential of MGs in remediation of wastewater contaminated with azo dyes. More MGs, e.g., Fe-, Mg-, MgZn- and Al-based ones, were subsequently applied to azo dye degradation^33^,^34^,^35^,^36^,^37^,^38^,^39^,^40^,^41^. The reacting rate was effectively enhanced, and the problem of mass loss was alleviated to a certain extent. Here, we report the application of Co-based MG to degrade azo dyes in an aqueous solution for the first time. Co-based MG exhibits better resistance to corrosion in wastewater due to its relatively higher standard reduction potential in aqueous solutions than Fe- and Mg-based MGs^42^. The ball-milled Co-based MG exhibited superior catalytic activity and durability as well as little mass loss when degrading the azo dye compared to reported Fe- and Mg-based MGs and their crystalline counterparts.

## Results

### Preparation and characterization of Co-based MG powders

**Figure 1:**
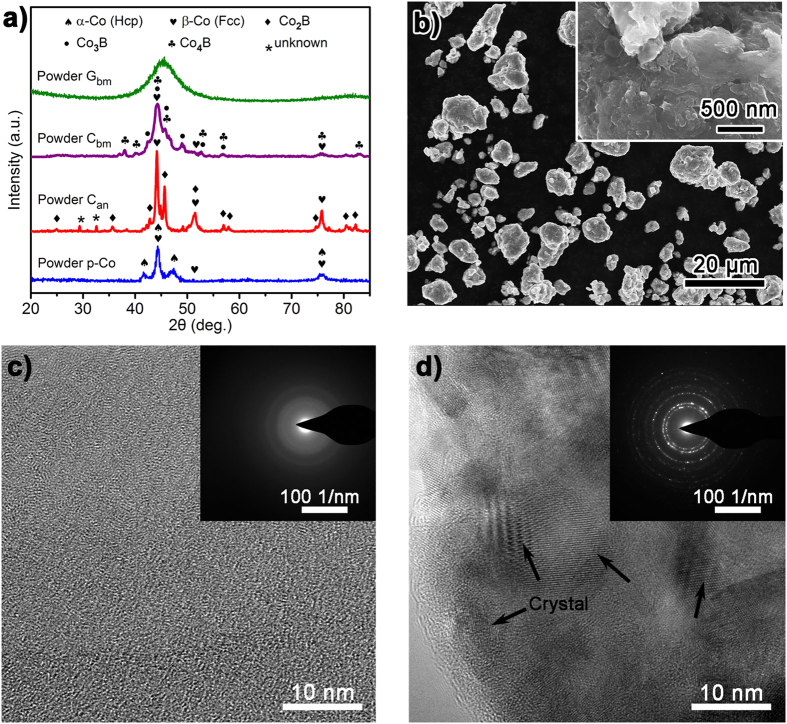
(**a**) XRD patterns of Powder G_bm_, Powder C_bm_, Powder C_an_ and Powder p-Co. (**b**) SEM images of the ball-milled Powder G_bm_. HRTEM images of (**c**) Powder G_bm_ and (**d**) Powder C_bm_, illustrating the amorphous and nanocrystalline structures obtained for Powder G_bm_ and Powder C_bm_, respectively.

Degradation of azo dyes by ZVMs is a surface-mediated process^43^. Ball milling can increase the surface area of powders, thus accelerating the degradation process^34^,^35^,^39^. Prior to ball milling, the MG ribbons were annealed at approximately the onset glass transition temperature (*T*_*g*_). Sufficient thermal relaxation cannot alter the amorphous structure (Fig. S1a) but embrittles the ribbons, thus facilitating the pulverization of the ribbons in the ball-milling process. Four powders were prepared: Powder G_bm_, Powder C_bm_, Powder C_an_ and Powder p-Co (details are provided in the Methods). Figure 1a presents the X-ray diffraction (XRD) patterns of Powder G_bm_, which exhibits broad diffusive diffraction peaks characteristic of an amorphous structure. Due to the metastable nature of the amorphous phase, high-energy input (i.e., an increase in the rotation speed or milling time) reduced its stability, inducing nanocrystallization of β-Co, Co_3_B and Co_4_B^40^, as indicated by the XRD patterns of Powder C_bm_ in Fig. 1a. The amorphous nature of Powder G_bm_ was further verified by differential scanning calorimetry (DSC, Fig. S1b) and transmission electron microscopy (TEM). Figure 1c shows a high-resolution TEM (HRTEM) image inset with selected area electron diffraction (SAED) patterns, which clearly demonstrate that the particles are fully amorphous either on the surface or inside. By contrast, in Powder C_bm_ (Fig. 1d), the surface layer, with a thickness of several nanometers, is amorphous, whereas the inside is composed of nanocrystals. Powder C_an_ and Powder p-Co, which correspond to Co-based nanocrystallized powders prepared by annealing Powder G_bm_ at 900 K for 120 min and pure Co nanocrystalline powders prepared by ball-milling the gas-atomized pure Co powders, respectively, are shown in Fig. 1a.

The morphology of the particles was characterized by scanning electron microscopy (SEM), which revealed that the particles had similar morphology for all powders (Fig. S2). Figure 1b shows the corresponding SEM images of Powder G_bm_. The particles have good separability. The surface exhibits high roughness and corrugation, which would significantly expand the active surface area and provide more active sites for the reaction to improve the degradation rate. The size distribution of the particles was 8 ~ 12 μm in median diameter (D50), as measured by a laser particle analyzer (Fig. S3). The specific surface area for Powder G_bm_ was approximately 0.24 m^2^ g^−1^ as determined by the Brunauer-Emmett-Teller (BET) method.

### Reactivity in the Acid Orange II aqueous solution

**Figure 2:**
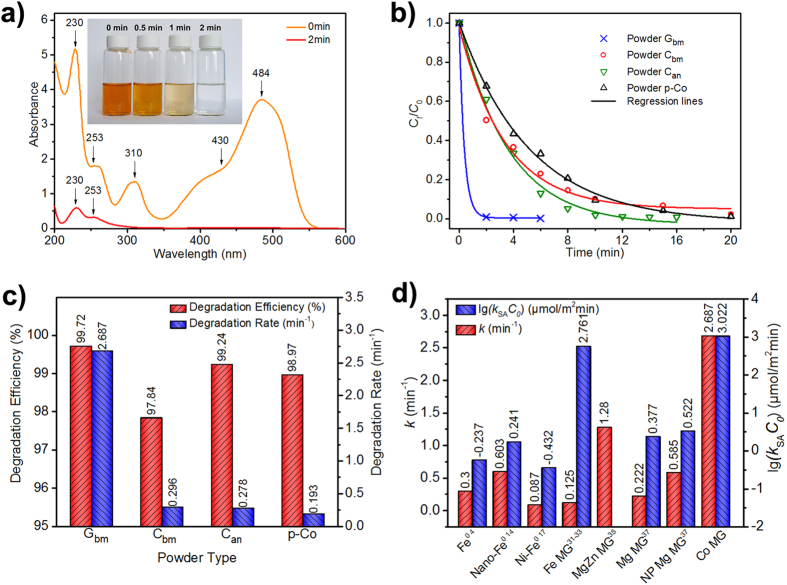
(**a**) UV-vis spectra of the AO II solution treated with Powder G_bm_ at room temperature as a function of reaction time, inset with a photograph of the AO II solution at different treating times. (**b**) The dependence of *C*_*t*_ normalized by *C*_*0*_ on reaction time for all four powders at room temperature. The solid lines are nonlinear fitting to the experimental data points. (**c**) Comparison of the AO II degradation efficiency and degradation rate for the four studied powders. (**d**) Comparison of the degradation capability of the present Co-based MG and other investigated ZVMs, confirming the ultrahigh reactivity of the Co-based MG in degrading the AO II solution.

Acid Orange II (AO II), a model compound of azo dye, was used to examine the degradation capabilities of the Co-based MG powders. UV-vis absorption spectra of AO II usually exhibit a strong absorption at λ_max_ = 484 nm originating from the conjugated structure formed by the azo bond (-N = N-); the intensity of this bond is proportional to the concentration of AO II in solution^3^. Figure 2a shows the change in the UV-vis absorption spectra of the AO II solution (the initial concentration of AO II was 0.2 g/L) treated with Powder G_bm_ as a function of reaction time. As the reaction progressed, the intensity of the absorption peak decreased due to degradation of the azo bond. In this study, the AO II solution treated by Powder G_bm_ changed from orange to colorless and transparent in less than 2 minutes, as shown in the inset of Fig. 2a. Complete degradation of the azo bond was also indicated by the intensity of the absorption peak (λ_max_ = 484 nm), which approached zero in the 2-minute spectra in Fig. 2a. These results demonstrate that Powder G_bm_ completely degraded AO II ultrafast in aqueous solution, approximately 30 times faster than the ball-milled Fe-based MG^34^ and several thousand times faster than the widely investigated Fe^0 4^.

To illustrate the structural effect, the degradation behaviors (Fig. S4) of Powder C_bm_, Powder C_an_ and Powder p-Co were compared with that of Powder G_bm_. The dependence of *C*_*t*_ normalized by *C*_*0*_ on the reaction time for all four powders is presented in Fig. 2b, where *C*_*t*_ and *C*_*0*_ are the real-time and initial concentrations of AO II in solution, respectively. When the structure was nanocrystalline rather than amorphous, the degradation capacity decreased, and the time required for complete degradation increased, for example, to 14 minutes for Powder C_bm_. These results suggest that an amorphous structure is more effective for AO II degradation. The decay behavior of *C*_*t*_/*C*_*0*_ with reaction time was fit well nonlinearly (Fig. 2b) by


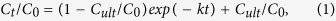


where *C*_*ult*_ is the ultimate residual dye concentration, *t* is the reaction time, and *k* denotes the empirical rate constant^15^. Thus, the degradation of AO II obeys a pseudo-first-order kinetic model. The parameters *k* representing the degradation rate and *η = 1 − C*_*ult*_*/C*_*0*_ defining the degradation efficiency^14^ can be derived by nonlinear fitting, as shown in Fig. 2c. Powder G_bm_ not only exhibits the highest empirical degradation rate constant of *k* = 2.687, more than an order of magnitude higher than the other three powders, but also achieves the highest degradation efficiency of *η = *99.72%. Thus, the Co-based MG powder exhibits superior performance in degrading the AO II solution.

Figure 2d compares the degradation capabilities of Powder G_bm_ and other investigated ZVMs. To take the reported differences in the surface areas of the ZVMs and initial concentrations of dyes into account, the parameter *k*_*SA*_*C*_*0*_ was calculated to compare the degradation capability^4^, where *k*_*SA*_ is the surface-area-normalized rate constant. Although the empirical rate constant of the amorphous alloys is not always higher than that of the crystalline ZVMs, *k*_*SA*_*C*_*0*_ is larger for MGs than for crystalline alloys, indicating MGs can support more active sites. The rate constant of the Co-based MG powder is higher than those of all investigated amorphous alloys: two times higher than that of MgZn-based MG^35^ and tens of times higher than those of Fe-based amorphous alloys^31^,^32^,^33^. The parameter *k*_*SA*_*C*_*0*_ is also highest for the present Co-based MG and is thousands of time higher than that of commercial Fe^0^ 3,4. As shown in Fig. 2d, the parameter *k*_*SA*_*C*_*0*_ was not determined for MgZn-based MG because the specific surface area was not reported^35^. Although the experimental parameters, such as the mass concentration of the particles and the initial concentration of dyes, were the same for both MgZn- and Co-based MGs, the density of MgZn-based MG was significantly lower than that of Co-based MG. Consequently, *k*_*SA*_*C*_*0*_ should be slightly smaller for MgZn-based MG. In short, Co-based MG may be the most efficient material for degrading azo dyes thus far.

In addition to high reactivity in the degradation process, the Co-based MG powder exhibits relatively low mass loss. We used inductively coupled plasma-atomic emission spectrometry (ICP-AES) to measure the total concentration of Co cations in the solution at different times when Co-based MG powder was exposed to the azo-dye aqueous solution (Fig. S5). The total concentration of Co cations remained constant at 2.8~4.5 mg L^−1^, thus indicating much lower mass loss than Fe- and MgZn-based MGs^31^,^32^,^33^,^34^,^35^. The mechanism of Co-based MG may differ from that of reported Fe- and MgZn-based MGs and Fe^0^, breaking the trade-off between reactivity and mass loss in popular ZVM techniques and making the application of Co-based MG of high commercial interest.

### Applicability of Co-based MG

**Figure 3:**
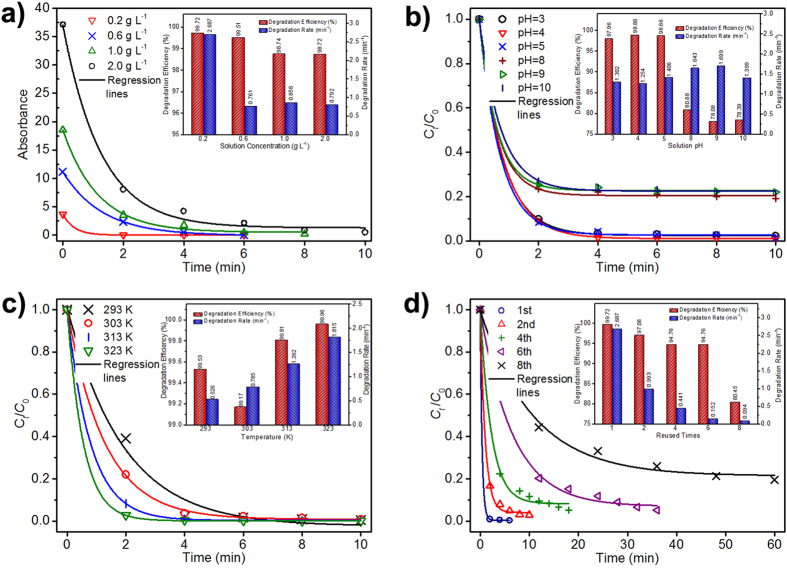
Influence of (**a**) the initial concentration of azo dye, (**b**) pH, and (**c**) environmental temperature on the degradation behaviors of AO II solutions, inset with the corresponding degradation rate and degradation efficiency, respectively. (**d**) The dependence of *C*_*t*_ normalized by C_0_ as a function of time for the different recycles of Powder G_bm_, inset with the corresponding degradation efficiencies and degradation rates for the different recycles.

To explore the applicability of Co-based MG, we examined the influence of working conditions, such as the initial concentration of azo dye, pH, and environmental temperature, on the degradation behaviors. As shown in Fig. 3a, when the concentration increased from 0.2 g L^−1^ to 2.0 g L^−1^, the degradation of AO II remained very fast and was nearly complete in 10 minutes. The parameters of *k* and *η* were obtained by nonlinear fitting, revealing a high degradation efficiency above 98% for all concentrations, as illustrated in the inset of Fig. 3a. Changes in AO II concentration in the experimental range slightly affected the degradation capability of Co-based MG powders. The influence of pH variation differed (Fig. 3b). In the examined pH range of 3~10, the degradation process was complete in a very short period of time, consistent with the fast degradation rate of this MG. However, high degradation efficiency of ~98% was maintained for pH values lower than 7, whereas the degradation efficiency decreased to ~78% for pH values larger than 7. The difference in the degradation efficiency induced by the increase in pH is probably attributable to a different degradation mechanism, which could be inferred from the different UV-vis absorption spectra of the products (Fig. S6) in solution with pH of 3~10.

To illustrate the influence of environmental temperature, we selected the AO II solution with a concentration of 2.0 g L^−1^ because the degradation reaction in the 0.2 g L^−1^ solution of AO II is too rapid. Figure 3c shows the decay behaviors of *C*_*t*_/*C*_*0*_ with reaction time at environmental temperatures of 293 K to 323 K. Increasing the environmental temperature enhanced the degradation capability of the Co-based MG powder, as indicated by the increase in *k* from 0.526 min^−1^ to 1.815 min^−1^ and *η* higher than 99%.

We further tested the durability of the present Co-based MG powders (Fig. 3d). As recycling increased, more time was needed to accomplish the degradation process. However, all degradation processes continued to obey the pseudo-first-order exponential decay kinetic model, as indicated by the good nonlinear fitting of the dependence of *C*_*t*_/*C*_*0*_ on reaction time with Equation (1). The obtained *k* and *η* are shown in the inset of Fig. 3d. A dramatic decrease in *k* from 2.687 min^−1^ to 0.993 min^−1^ occurred on the second use. After the first use, we examined the morphology of the Co-based MG powders by SEM. Products partially covered the surface of the powders, directly reducing the reaction sites for the second use and correspondingly lowering *k*. By the sixth use, *k* declined to 0.152 min^−1^, i.e., two orders of magnitude. However, *η* remained above 94%. On the eighth use, the degradation capability further deteriorated, as indicated by *k* = 0.094 min^−1^ and *η* = ~80%. These results demonstrate that the present Co-based MG is very durable. XRD measurements (Fig. S7) revealed that the Co-based MG powders still retained the amorphous structure after the eighth use.

## Discussion

**Figure 4:**
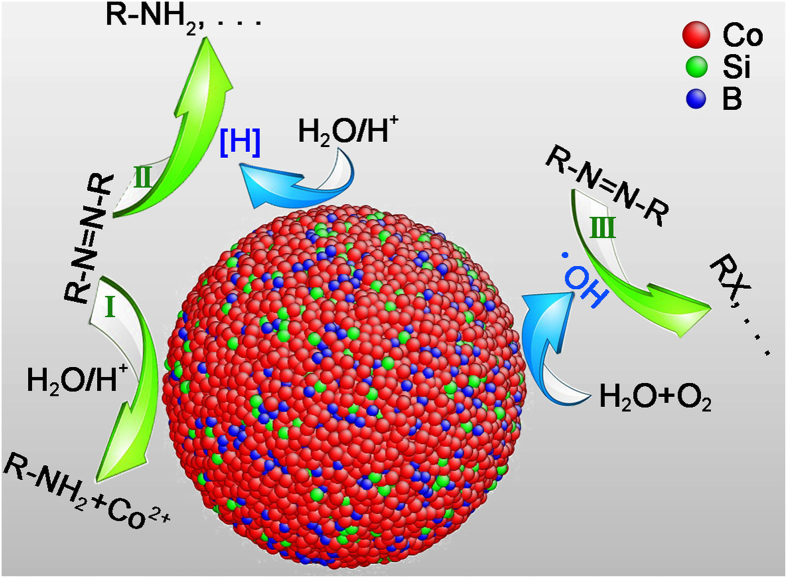
Illustration (drawn by X.D.Q.) of the major reactions occurring in the present system and the mechanisms of AO II degradation.

The prevailing degradation mechanism of azo dyes in the ZVM-H_2_O system can be summarized as follows^6^,^8^,^43^: (i) reductive degradation of azo dyes with direct electron transfer from ZVM at the surface of ZVM; (ii) degradation of azo dyes by reaction with dissolved M(II) or H/H_2_, which are products of ZVM corrosion; and (iii) catalytic hydrogenation. All pathways are presumably coupled with the electrochemical corrosion of metals^6^,^8^. Take Fe^0^ as an example. The corrosion of Fe^0^ governs the rate of contaminant removal by Fe^0^ 8,9. Consequently, methods that accelerate Fe^0^ corrosion can be used to enhance contaminant sequestration by Fe^0^ 8,9. This strategy was not applicable in this study because Co-based MG powders exhibited the highest reaction rate of all investigated powders, as shown in Fig. 2, and amorphous alloys usually possess higher corrosion resistance than their crystalline counterparts^26^,^27^, as evidenced by the low mass loss in the present study. The ultrafast degradation of azo dyes by Co-based MG powders is attributable to their amorphous structure. Figure 4 illustrates the possible degradation reactions and mechanisms occurring in the present system.

At the atomic level, Co-Si-B MG can be described as a space-filling network of short-range ordering polyhedra with a scale of several angstroms to several nanometers^44^. The atoms are in the highly coordinatively unsaturated state. More atoms with unsaturated coordination are present on the surface of the amorphous structure than the crystalline one. The role of these atoms is similar to that of Pd atoms in bimetallic Pd-Fe^0^ and Pd-Mg^0^ particles, in which they function as catalysts and the absorbed atomic hydrogen species are responsible for hydrogenation degradation of azo dyes^10^,^16^,^17^,^18^,^19^. Thus, these atoms obviously contribute to the increase in the reaction rate. This mechanism predominates the degradation process of azo dyes and is also supported by the following results in the present study. First, if this pathway predominates the reductive cleavage of the -N = N- bond, the cobalt content should be at least 67.290 mg L^−1^ as quantitatively calculated by mass balance. In fact, the cobalt content in the solution was merely 2.8~4.5 mg L^−1^ as determined by ICP-AES after the reaction (Fig. S5), which is far below that calculated for degrading the azo dye by direct transfer of electrons from MG to the dye molecules. Second, the activation energy for the degradation reaction of AO II was evaluated by the Arrhenius-type relationship *lnk*_*T*_* = lnA* − *E*_*a*_*/RT*, where *k*_*T*_ is the empirical rate constant at the thermodynamic temperature *T*, *A* is the pre-exponential factor, *E*_*a*_ is the activation energy, and *R* is the molar gas constant. *E*_*a*_ was approximately 33 kJ mol^−1^ over the temperature range of 293 K to 323 K (Fig. S8), much lower than that for Fe^0^ 34 and Fe- and MgZn-based MGs^34^,^35^. These results support the catalytic effect and the transfer of active hydrogen to the azo dye molecules to facilitate the cleavage of the -N = N- bond.

Moreover, the Co-based MG contains metalloid elements of Si and B as high as 22 at.%, which not only enhances glass-forming ability, thus facilitating the preparation of the Co-based MG powder, but may also promote the degradation process through the formation of local galvanic couples between Si, B and Co^42^. The role of local galvanic couples is controversial, and it is generally accepted that they could benefit the oxidizing degradation of azo dyes^8^,^9^,^10^. Hydroxyl radicals produced by the reaction of water and dissolved oxygen electro-catalyzed on the surface of Co-based MG would have a high oxidation potential of ~2.8 V and oxidatively decompose the azo dye^18^.

The amorphous structure is not the equilibrium state and is energetically higher than the corresponding crystalline state; consequently, MGs are always more active than their crystalline counterparts. The degradation reaction can occur on more sites of the MG surface, thus improving the reaction rate. We observed the morphologies of particles exposed in aqueous solution for 5 min for all investigated materials (Fig. S2). Although no obvious difference was observed among the four types of particles before reaction, a significant difference was observed after 5 min exposure. The surface of MG particles was quickly coated with the reaction products uniformly and fluffily, whereas sparse reaction products were observed on the surface of the other types of particles^32^,^34^. This result implies that the amorphous structure supports more reactive sites than the crystalline structure, which undoubtedly favors an increase in the reaction rate for a surface-mediated degradation process^43^. The precipitated reaction products will also enhance the absorption of the dye molecules because of the porous structure^8^. Consequently, the concentration of azo dye increased in the vicinity of the surface of the particles, likewise accelerating the degradation reaction.

In summary, Co-based MG was applied to degrade azo dyes for the first time. The Co-based MG exhibits superior properties, such as high reactivity and efficiency, wide applicability, good reusability and low corrosion loss. The degradation process can be described by a pseudo-first-order kinetic model. Surface catalysis plays a significant role in the degradation process, which contributes to the ultrafast degradation rate. The effects of Co-based MG should remain similar to those of Fe^0^ technology when treating other environmental contaminants. From this perspective, Co-based MG particles may be an ideal ZVM technology to sequestrate environmental contaminants^9^. These findings will promote the practical application of MGs as functional materials.

## Methods

### Preparation of Co-based MG powders

Metallic glass ribbons with a nominal composition of Co_78_Si_8_B_14_ in atomic percentage were prepared by vacuum melt-spinning. Then, the ribbons were annealed (673 K, 5 h) below the crystallization temperature according to the DSC pattern shown in Fig. 1a under an argon atmosphere. After annealing embrittlement, the ribbons were cut into pieces and subjected to high-energy ball milling. The mill pots containing ribbon pieces and steel ball bearings were pumped to a high vacuum and then filled with high-purity argon gas for protection. Powder G_bm_ and Powder C_bm_ were manufactured by ball milling the glassy ribbon pieces for 40 h at a speed of 250 r min^−1^ and for 34 h at 400 r min^−1^, respectively. Powder C_an_ was obtained by vacuum annealing Powder G_bm_ at 900 K for 120 min. Powder p-Co was prepared by ball milling the gas-atomized pure Co powders for 40 h at 250 r min^−1^.

### Physical characterization

The structure of the powders was verified by DSC, XRD and TEM. The diameter and the morphology of the particles were characterized using a laser particle analyzer and by SEM, respectively. The BET surface area was determined by N_2_ absorption/desorption analysis conducted at 77 K.

### AO II degradation characterization

The AO II aqueous solution (0.2 g L^−1^) was prepared by dissolving Acid Orange II at the desired concentration in distilled water to prepare 1 L of solution. Degradation experiments were conducted in a reactor (100 mL) fastened in a reciprocating shaker. The shaker was timed starting at 360 r min^−1^ when the powders (0.12 g) and Acid Orange II aqueous solution (20 mL) were injected into the reactor. Periodically, 3 mL of solution was withdrawn and used to measure the concentration of AO II after centrifugation. The concentration of the AO II solution was analyzed by UV-vis spectroscopy (Model V-550JAS.CO., America) at a maximum wavelength of 484 nm, corresponding to the azo bond (-N = N-). After the reaction, the collected powders washed of reagents were again studied by SEM to examine the change in surface morphology. In addition, the cobalt content in the AO II aqueous solution after reaction was determined by ICP-AES. The powders after reaction were collected and washed of reagents, then cleaned of products coated on the surface using HCl (0.1 M); finally, the powders were washed with ethyl alcohol at least three times. After drying, the powders were applied in the degradation process a second time, and the same procedure was repeated until the degradation efficiency decreased radically. The effects of temperature, solution acidity, solution concentration and particle dosage on the degradation process were also examined. All experiments were conducted at room temperature (~25 °C) except those studying the effect of temperature. For experiments studying the effect of temperature, the solution temperature remained at 20, 30, 40 and 50 °C, respectively. Thus, the influence of temperature on the degradation rate was investigated. We also adjusted the solution acidity with pH values of 3, 4, 5, 8, 9 and 10 by adding HCl (0.1 M) or NaOH (0.1 M) to observe the influence of the pH value on the degradation rate. AO II solutions with concentrations of 0.2 g L^−1^ ~ 2.0 g L^−1^ were used in the degradation process.

## Additional Information

**How to cite this article**: Qin, X. D. *et al.* Ultrafast degradation of azo dyes catalyzed by cobalt-based metallic glass. *Sci. Rep.* **5**, 18226; doi: 10.1038/srep18226 (2015).

## Supplementary Material

Supplementary Information
