# Supplementary material for: Ultrafast degradation of azo dyes catalyzed by cobalt-based metallic glass
Source: Sci Rep. 2015 Dec 14;5:18226. doi: 10.1038/srep18226 (PMC4677396; doi:10.1038/srep18226)
Supplement: Supplementary Information [file srep18226-s1.doc]

**Supplementary figures：**

**Ultrafast degradation of azo dyes catalyzed by cobalt-based metallic glass**

X. D. Qin^1,2^, Z. W. Zhu^1,*^, G. Liu^1^, H. M. Fu^1^, H. W. Zhang^1^, A. M. Wang^1^, H. Li^1^, H. F. Zhang^1,*^

^1^Shenyang National Laboratory for Materials Science, Institute of Metal Research, Chinese Academy of Sciences, Shenyang. 110016, China

^2^University of Chinese Academy of Sciences, Beijing. 100049, China

^*^Correspondence and requests for materials should be addressed to Z.W.Z. and H.F.Z. (email: zwzhu@imr.ac.cn and hfzhang@imr.ac.cn).


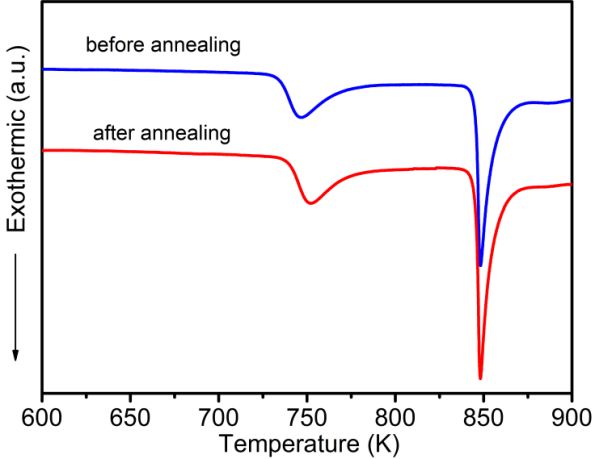


**a)**

**b)**


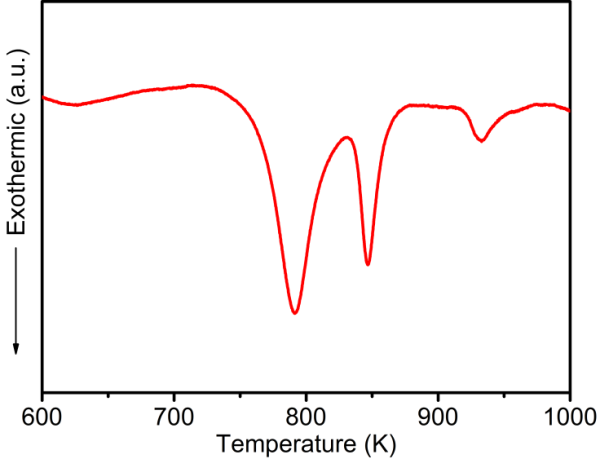


**Supplementary Figure 1.** DSC curves of (a) MG ribbons before and after annealing and (b) ball-milled Co-based MG powders (Powder G_bm_).


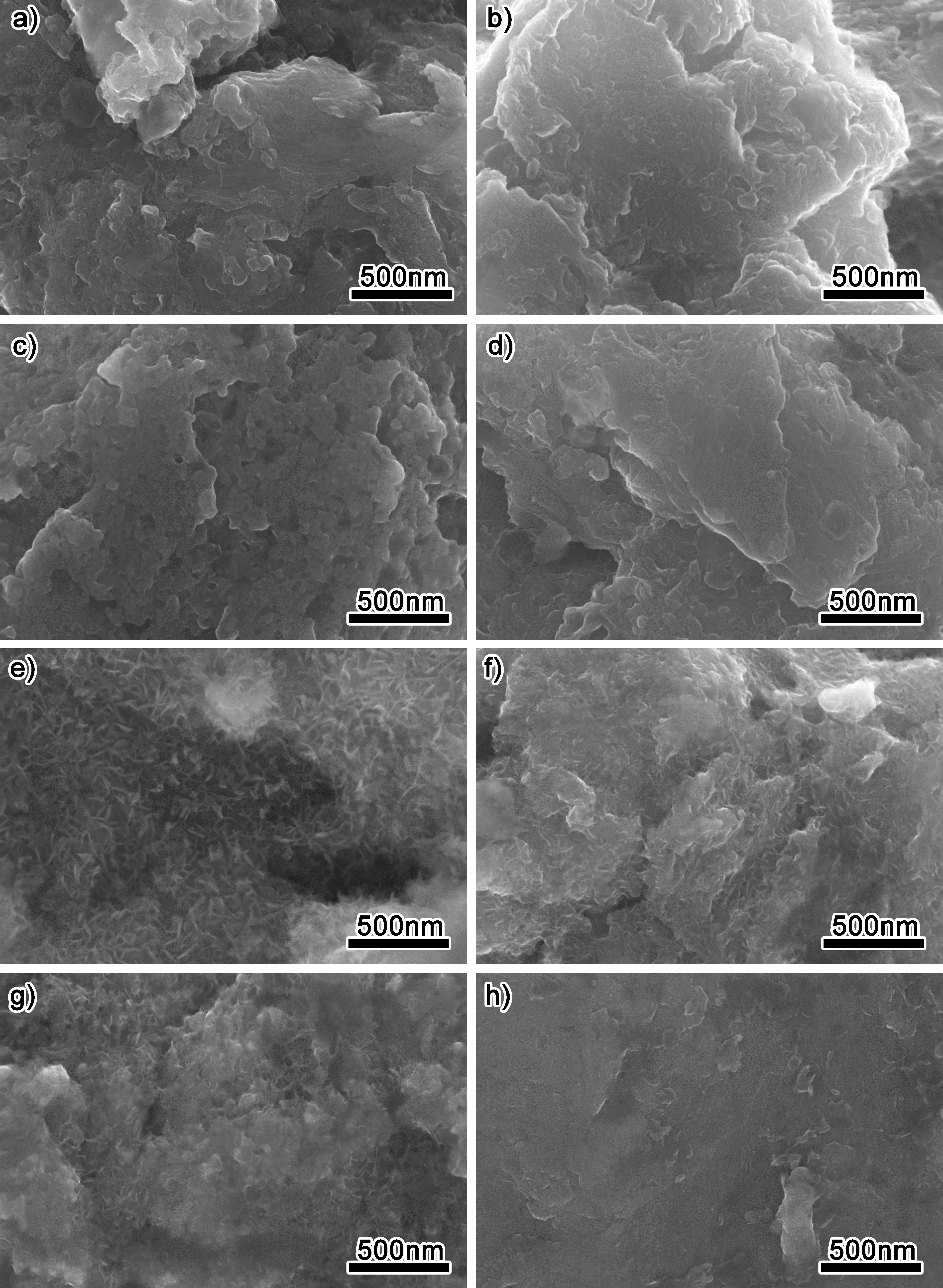


**Supplementary Figure 2.** Surface morphology of Powders (a) G_bm_, (b) C_bm_, (c) C_an_ and (d) p-Co before reaction and Powders (e) G_bm_, (f) C_bm_, (g) C_an_ and (h) p-Co after reaction.


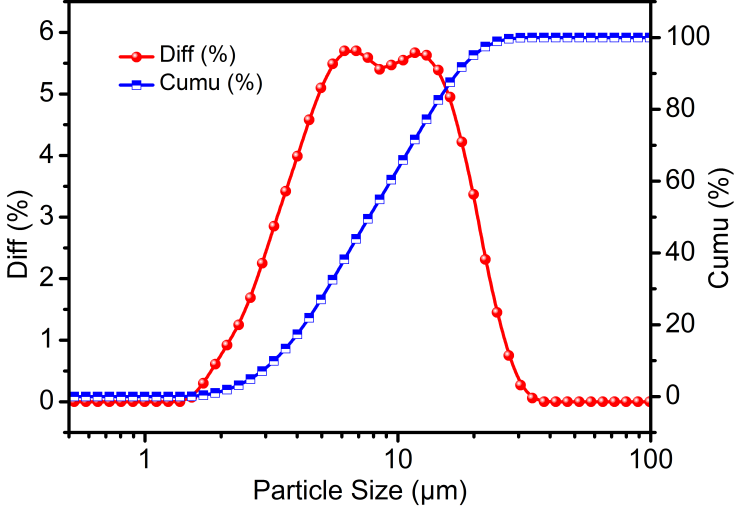

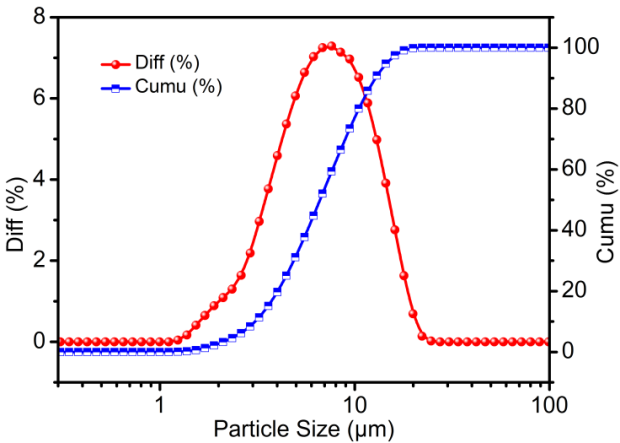

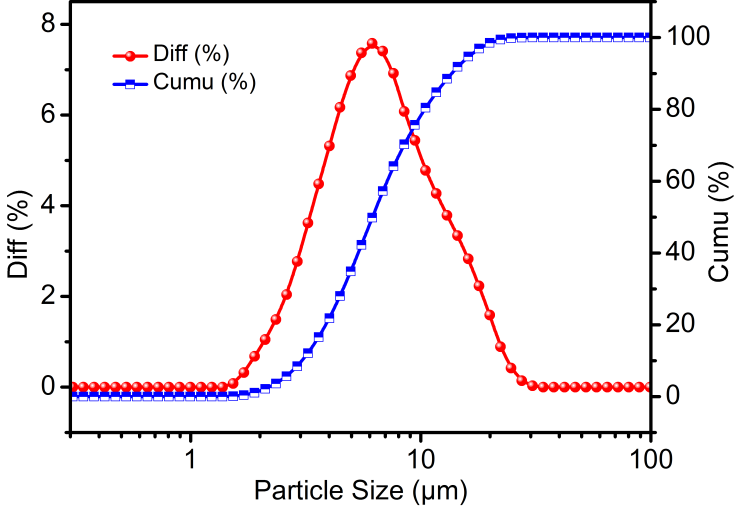

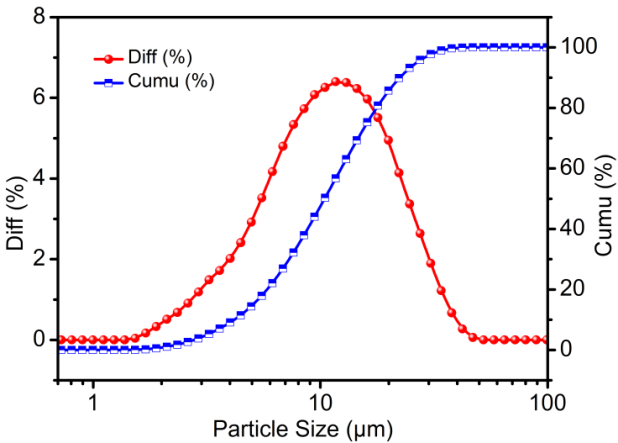


**a)**

**b)**

**d)**

**c)**

**Supplementary Figure 3.** Distribution of particle size for (a) Powder G_bm_, (b) Powder C_bm_, (c) Powder C_an_ and (d) Powder p-Co. The size distribution of the particles was 8~12 μm in median diameter (D50) for all four powders.

**a)**

**b)**

**c)**


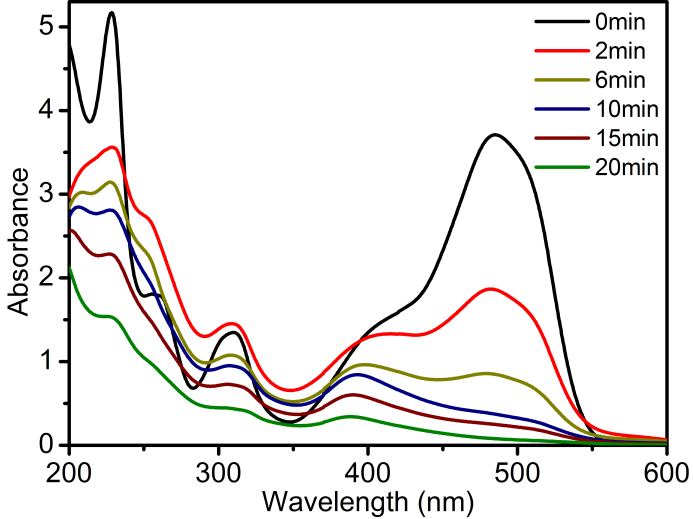

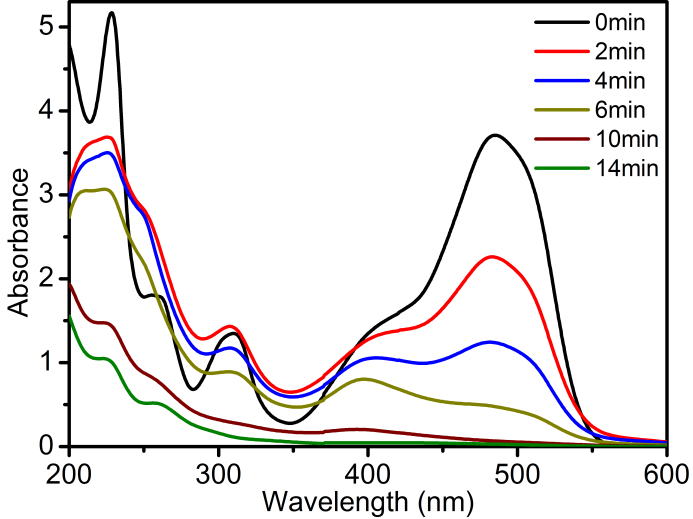

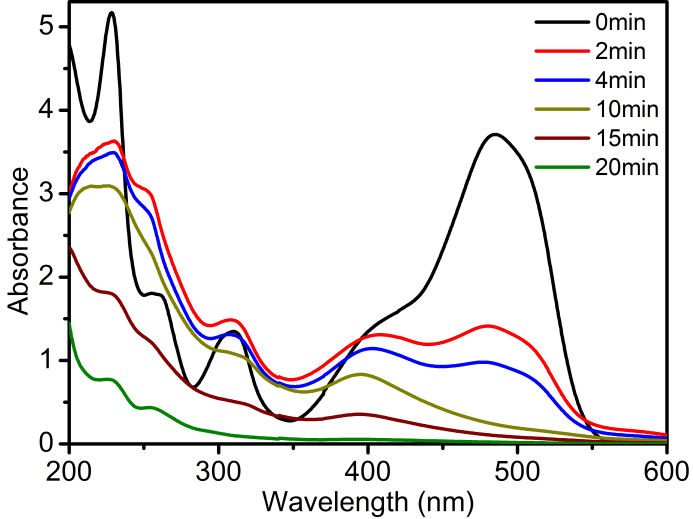


**Supplementary Figure 4.** UV-vis spectra of the AO II solution treated by (a) Powder C_bm_, (b) Powder C_an_ and (c) Powder p-Co at room temperature as a function of reaction time.

**
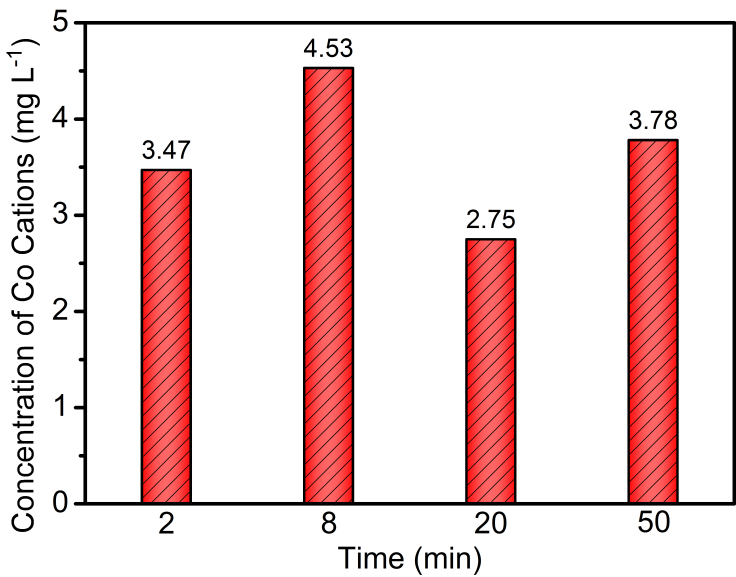
**

**Supplementary Figure 5.** Total concentration of Co cations in the AO II solution degraded by Powder G_bm_ as a function of reaction time.


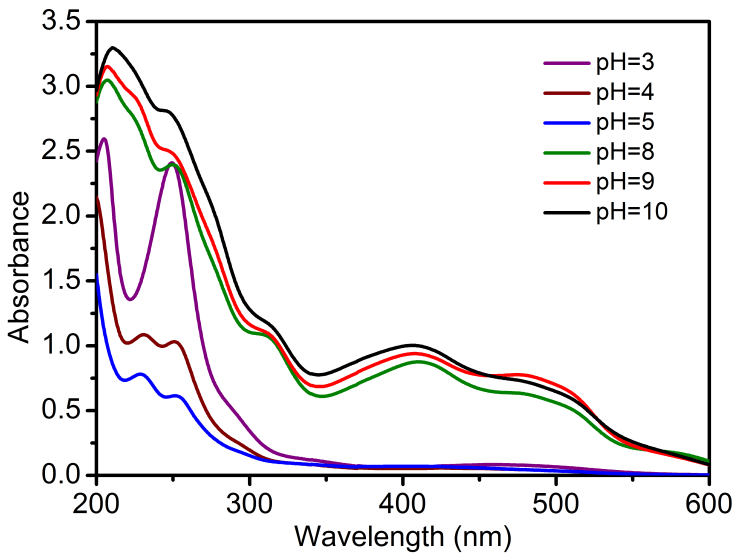


**Supplementary Figure 6.** UV-vis spectra of the Acid Orange II solution degraded by Powder G_bm_ for 30 min at different pH values. The reaction products differ at varying pH in the range of 3~10.


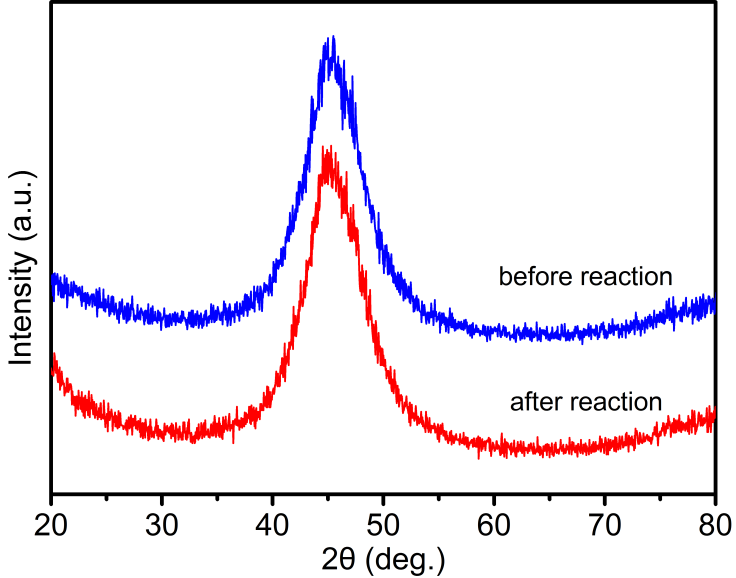


**Supplementary Figure 7.** XRD patterns of the ball-milled Powder G_bm_ before the first use and after the eighth use.


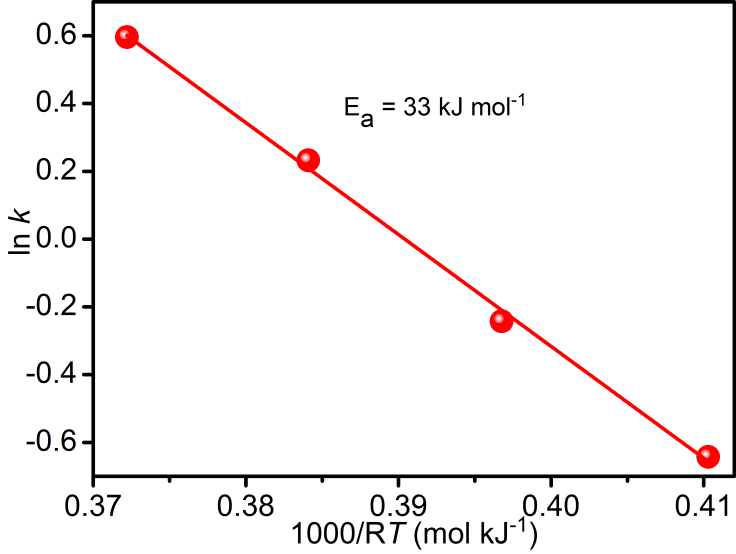


**Supplementary Figure 8.** Plot of ln *k* versus 1000/R*T*, the solid line is the fitting by Arrhenius equation to yield the activation energy.
